# The association between race and survival in glioblastoma patients in the US: A retrospective cohort study

**DOI:** 10.1371/journal.pone.0198581

**Published:** 2018-06-21

**Authors:** Andrew Bohn, Alexander Braley, Pura Rodriguez de la Vega, Juan Carlos Zevallos, Noël C. Barengo

**Affiliations:** Department of Medical and Health Science Research, Herbert Wertheim College of Medicine, Florida International University, Miami, Florida, United States of America; University of Kentucky, UNITED STATES

## Abstract

**Background:**

Glioblastoma is the most common primary brain cancer in adults with an incidence of 3.4 per 100,000, making up about 15% of all brain tumors. Inconsistent results have been published in regard differences in survival between white and black glioblastoma patients. The objective of this to study the association between race and in Glioblastoma patients in the USA during 2010–2014.

**Methods and findings:**

The National Cancer Institute’s Surveillance Epidemiology and End Results (SEER) database were used to evaluate race/ethnicity (White non-Hispanic, Black non-Hispanic, Asian/Pacific Islanders non-Hispanic (API)) and Hispanic) adults patients with first-time diagnosis of glioblastoma (International Classification of Diseases for Oncology, 3rd Edition [ICD-O-3], codes C711-C714, and histology type 9440/3) from 2010–2014. The primary outcome was 3-year overall survival which was defined as months from diagnosis to death due to any cause and cancer, Kaplan-Meier (KM) and log-rank test were used to compare overall survival times across race groups. Cox proportional hazard models were used to determine the independent effect of race on 3-year survival. Age, gender, health insurance coverage, primary site, tumor size, extent of surgery and year of diagnosis were included in the adjusted model. The 3-year overall survival for API-non Hispanic (NH) patients decreased by 25% compared with White NH glioblastoma patients (hazard ratio (HR) 0.75; 95% confidence interval (CI) 0.62–0.90)) after adjusting for age, gender, health insurance, primary site, tumor size, and extent of the surgery. Black NH (HR 0.95; 95% CI 0.80–1.13) and Hispanic (HR 1.01, 95% CI 0.84–1.21) exhibited similar mortality risks compared with White NH patients.

**Conclusion:**

Compared with White NH, API NH with glioblastoma have a better survival. The findings from this study can help increase the accuracy of the prognostic outlook for white, black and API patients with GBM.

## Introduction

Glioblastoma (GBM) is the most common primary brain cancer in adults with an incidence of 3.4 per 100,000, making up about 15% of all brain tumors [1, 2]. Despite advances in surgical and radiotherapy techniques the prognosis of GBM is dismal with a median survival of 12–14 months [1–3]. The current standard of care involves surgical resection along with temozolomide and radiotherapy, which has been shown to improve survival [4].

Racial disparities in treatment and subsequent survival of brain tumors has been documented in several previous studies [5–15]. However, large population-based analysis including all age and race groups in the US population is scant [5, 7–10]. Whereas some of them included all types of primary brain tumors [5, 9], others reported their findings specifically for GBM patients [7, 8, 10]. Moreover, the adjustments used in the analysis of previous studies varied a lot [5, 7–10]. Only one of them included a measure of socio-economic status in the statistical models [5]. In addition, only a few studies have included Asian or Hispanic glioblastoma patients and the results are inconsistent [7, 9, 10]. Finally, no study has investigated the association between race and survival in GBM patients using the recent information of the Surveillance Epidemiology and End Results (SEER) database during the past years.

The aim of this study was to investigate the association between race/ethnicity and survival in glioblastoma patients in the USA from 2010 to 2014.

## Material and methods

### Study design and setting

We conducted a secondary data analysis of the Surveillance Epidemiology and End Results (SEER) database. SEER is a cancer surveillance program of the National Cancer Institute’ s (NCI) that collects cancer incidence and survival data since 1973, including the Atlanta, Detroit, San Francisco-Oakland, Seatle-Puget Sound metropolitan areas and the states of Connecticut, Hawaii, Iowa, New Mexico, and Utah [16].

### Study participants

The population of interest included adults 18 years-of-age or older, identified as White non-Hispanic (NH), Black NH, API NH, and Hispanics with a first-time diagnosis of glioblastoma (International Classification of Diseases for Oncology, 3rd Edition [ICD-O-3], codes C711-C714, and morphology code 9440/3 from 2010 to 2014 (n = 3,517). The period of this study was chosen as in 2009 a new drug, bevacizumab, was approved for use in GBM. Treatment of bevacizumab may improve general survival after 2009. Patients who were reported only on death certificate or at autopsy (n = 13) or with no information on race (n = 15) or defined as American Indian /AK Native (n = 16) were excluded. Also people with survival time zero were excluded (n = 12). The final population consisted of 3,473 patients.

### Main variables

The primary outcome was overall survival (OS) which was defined as the time in months from diagnosis to death due to any cause and cancer. Patients who were alive at the date of the last contact were censored.

The race and ethnicity categories were limited to non-Hispanic White, Black or API, and Hispanic due to the small numbers for other races. Confounding variables evaluated included age at diagnosis, gender, insurance status, primary site, tumor size, and extent of the surgery. Age in years was collapsed into dichotomous categories (</ = 50, >50). Health insurance status was grouped into uninsured, any Medicaid and insured (combining SEER “Insured and Insured/No specifics” categories). Primary tumor sites were defined using ICD-O-3 topology codes into frontal, temporal, parietal, and occipital site (C711–C714 respectively). Tumor size was dichotomized using the tumor median size of 4.5 cm (<4.5,>/ = 4.5) and patients with unknown or not stated tumor size were included. Extent of the primary surgery was defined as “No Surgery”; “No Gross Total Resection” (No GTR) (this category included codes for local tumor destruction not otherwise specified (NOS), biopsy, surgery NOS, partial resection NOS, and subtotal resection; and “Gross Total Resection” (GTR) (this included gross total resection or radical total resection of the tumor). As the information used in this study was limited to the first course of treatment, such as radiation or chemotherapy status were not included in the analysis.

### Statistical analysis

All data accessed from SEER was de-identified (fully anonymized) and without any of the 18 direct identifiers according to the Health Insurance Portability and Accountability Act. The study population was characterized using descriptive statistics, these included examining frequency distributions of the categorical variables and assessing for missing data. Chi-square tests were used to compare categorical data (demographic and clinical characteristics among race/ethnicity categories). Analysis of variance was used for the age at diagnosis in years. Kaplan-Meier survival analysis was used to asses overall survival. The log-rank test was used to assess differences between survival curves across race/ethnicity. Univariate and Multivariate Cox Proportional hazard ratios (HRs) and 95% confidence intervals (CIs) to determine the independent effect of race/ethnicity and overall 3-year survival rates were calculated. The correlation matrix of all potential confounders were examined to check for multicollinearity in the adjusted model. Multivariate analysis was performed using Cox proportional hazards model to determine the independent effect of race/ethnicity on 3-year overall survival, hazard ratios and their respective 95% confidence intervals were calculated. The proportional hazard assumptions were tested graphically. Age, gender, health insurance status, primary site, tumor size and extent of surgery were included in the adjusted model. All p values reported are two-tailed, and a p-value of <0.05 was considered as statistically significant. Statistical analysis was performed using STATA (14.0, StataCorp, College Station, TX).

### Ethical considerations

Permission to use and access to the SEER database was obtained through the SEER website. Ethical approval was waived since the analysis was considered nonhuman subjects research by the Florida International University Health Sciences IRB.

## Results

Among the glioblastoma patients identified, the majority were white (83.2%) and 5.9%, 5.4%, and 5.5% were black NH, API NH and Hispanic respectively. Table 1 presents the baseline characteristics of patients in the US between 2010 and 2014 according to race/ethnicity. We identified significant racial/ethnicity differences in the age at diagnosis, health insurance, and extent of the primary surgery. The mean age at diagnosis was higher for white Non-Hispanic patients compared to non-Hispanic black, and Hispanics (64.4 vs.61.4, and 60.7, p = 0.015, respectively). Blacks NH (23.4%), Hispanics (22.9%) and API NH (18.8%) tended to be younger compared to (whites NH 13.8%). White NH patients were more frequently insured (91.3%), compared to 77.8% of blacks NH, 76.2% of API NH and 76.1% of Hispanic, (p<0.001). White NH and Black NH patients had a higher proportion of gross total resection surgery (37.6% and 37.1% respectively) compared to API NH (25.3%) and Hispanic (24.0%), while API NH had a higher proportion of no surgery (25.7%, p<0.001). Glioblastoma White, Black and Hispanic patients are more likely to die during the first three years after diagnosis (72.3%, 71.2% and 67.7% respectively) compared to API’s (63.4%, p = 0.040). There were not statistically significant difference among race/ethnicity in regards to gender (p = 0.728), primary site (p = 0.208), and tumor size (p = 0.356).

**Table 1:** Baseline characteristics of glioblastoma patients in the US, SEER from 2010 to 2014 according to race/ethnicity.

	Race/Ethnicity								
Characteristics	White NH^a^ (N = 2,890)		Black NH (N = 205)		API NH^b^ (N = 186)		Hispanic (N = 192)		p-value
	n	%	n	%	n	%	n	%	
**Age (years)**									<0.001
≤ 50	380	13.2	48	23.4	35	18.8	44	22.9	
> 50	2,510	86.9	157	76.6	151	81.2	148	77.1	
**Gender**									0.707
Male	1,681	58.2	118	57.6	105	56.5	119	62.0	
Female	1,209	41.8	87	42.4	81	43.6	73	38.0	
**Insurance**									<0.001
Uninsured	61	2.2	12	5.9	9	4.9	15	8.0	
Any Medicaid	185	6.5	33	16.3	35	18.9	30	16.0	
Insured	2,587	91.3	158	77.8	141	76.2	143	76.1	
**Primary Site**									0.400
Frontal	1,111	38.4	65	31.7	80	43.0	72	37.5	
Temporal	996	34.5	83	40.5	57	30.7	65	33.9	
Parietal	619	21.4	46	22.4	42	22.6	40	20.8	
Occipital	164	5.7	11	5.4	7	3.8	15	7.8	
**Tumor Size (cm)**									0.306
< 4.5	1,223	42.3	89	43.4	72	38.7	71	37.0	
> = 4.5	1,250	43.3	89	43.4	94	50.5	95	49.5	
Unknown	417	14.4	27	13.2	20	10.8	26	13.5	
**Extent of surgery**									<0.001
No Surgery	435	15.1	29	14.2	48	25.8	46	24.0	
No GTR^c^	1,366	47.4	100	48.8	91	48.9	100	52.1	
GTR	1,084	37.6	76	37.1	47	25.3	46	24.0	
**Three-year survival status**									0.044
Alive	802	27.8	59	28.8	68	36.6	62	32.3	
Dead	2,088	72.3	146	71.2	118	63.4	130	67.7	

^a^Nonhispanic

^b^Asian Pacific Islander

^c^Gross Total Resection

**Fig 1:**
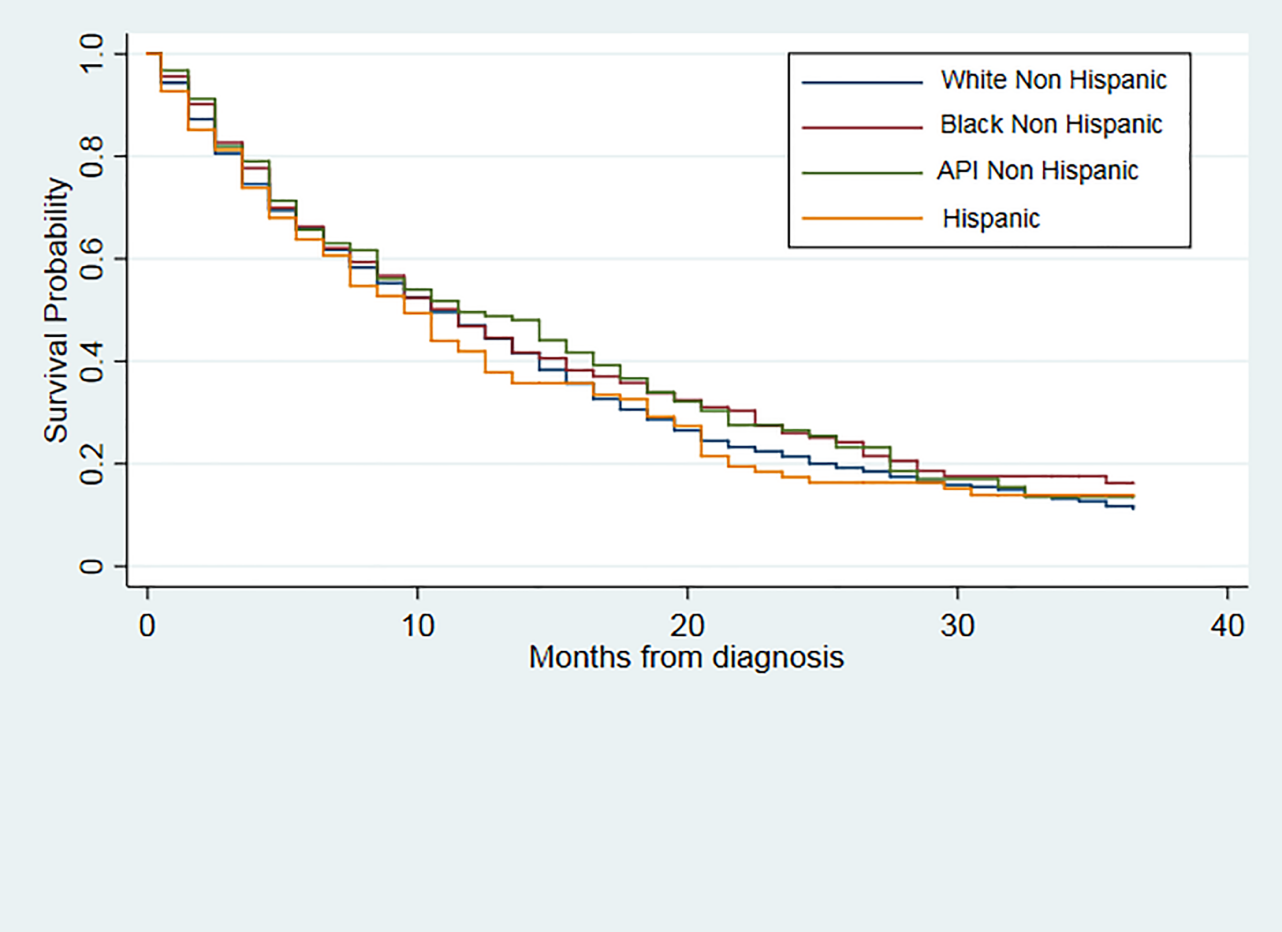
Unadjusted Kaplan Meier estimates of 3-year overall survival of glioblastoma patients by race/ethnicity.

Fig 1 shows the Kaplan Meier curves for the 3-year overall survival in months by race/ethnicity. The log-rank test revealed no statistically difference (p-value<0.370) among race/ethnicity groups, with a median survival times for Whites NH (10.5 months; 95% CI 10.5–11.5), Blacks NH (11.5 months; 95% CI 8.5–13.5), API’s NH (11.5 months; 95% CI 8.5–15.5) and Hispanic (9.5, 95% CI 7.5–11.5).

The unadjusted Cox regression model suggested no differences between Blacks NH (HR 0.90; 95% CI 0.76–1.07), API NH (HR 0.90; 95% CI 0.74–1.08) and Hispanic (HR 1.04; 95% CI compared to White NH glioblastoma patients (Table 2). The correlation matrix of all potential confounders in the adjusted model were examined and no multicollinearity was found. After adjusting for age, gender, health insurance status, primary site, tumor size and extent of the surgery, the risk of death at 3-years post diagnosis among API’s (HR 0.75; 95% CI 0.62–0.90) decreased significantly compared to White NH. Older patients, with greater tumor size (> = 4.5 cm) or unknown tumor size, with no surgery or no GTR were significantly and independently associated with poorer prognosis.

**Table 2:** Unadjusted and adjusted hazard ratios between race/ethnicity and 3-year overall survival in glioblastoma patients in the US, SEER from 2010–2014.

	Unadjusted		Adjusted	
	HR^a^ (95% CI^b^)	p-value	HR (95% CI)	p-value
**Race/Ethnicity**				
White NH^c^	Ref^d^		Ref	
Black NH	0.90 (0.76–1.07)	0.225	0.95 (0.80–1.13)	0.585
API^e^ NH	0.90 (0.74–1.08)	0.246	0.75 (0.62–0.90)	0.003
Hispanic	1.04 (0.87–1.24)	0.692	1.01 (0.84–1.21)	0.942
**Age (years)**				
≤ 50	Ref		Ref	
> 50	2.35 (2.07–2.67)	<0.001	2.18 (1.91–2.49)	<0.001
**Gender**				
Male	Ref		Ref	
Female	1.09 (1.01–1.18)	0.033	1.07 (0.99–1.16)	0.081
**Insurance**				
Uninsured	0.82 (0.64–1.05)	0.122	0.99 (0.77–1.28)	0.926
Any Medicaid	1.05 (0.91–1.22)	0.486	1.06 (0.92–1.23)	0.401
Insured	Ref		Ref	
**Primary Site**				
Frontal	Ref		Ref	
Temporal	0.93 (0.85–1.02)	0.145	0.96 (0.87–1.05)	0.382
Parietal	1.10 (0.99–1.23)	0.063	1.04 (0.93–1.15)	0.507
Occipital	0.95 (0.80–1.13)	0.536	0.95 (0.80–1.14)	0.601
**Tumor Size (cm)**				
< 4.5	Ref		Ref	
> = 4.5	1.06 (0.97–1.15)	0.198	1.11 (1.02–1.21)	0.020
Unknown	1.15 (1.20–1.35)	0.003	1.19 (1.06–1.35)	0.004
**Extent of surgery**				
No Surgery	3.52 (3.14–3.95)	<0.001	3.33 (2.96–3.75)	<0.001
No GTR^f^	1.42 (1.30–1.55)	<0.001	1.43 (1.30–1.56)	<0.001
GTR	Ref		Ref	

^a^Hazard ratio

^b^Confidence interval

^c^Nonhispanic

^d^Reference group

^e^Asian Pacific Islander

^f^Gross Total Resection

**Fig 2:**
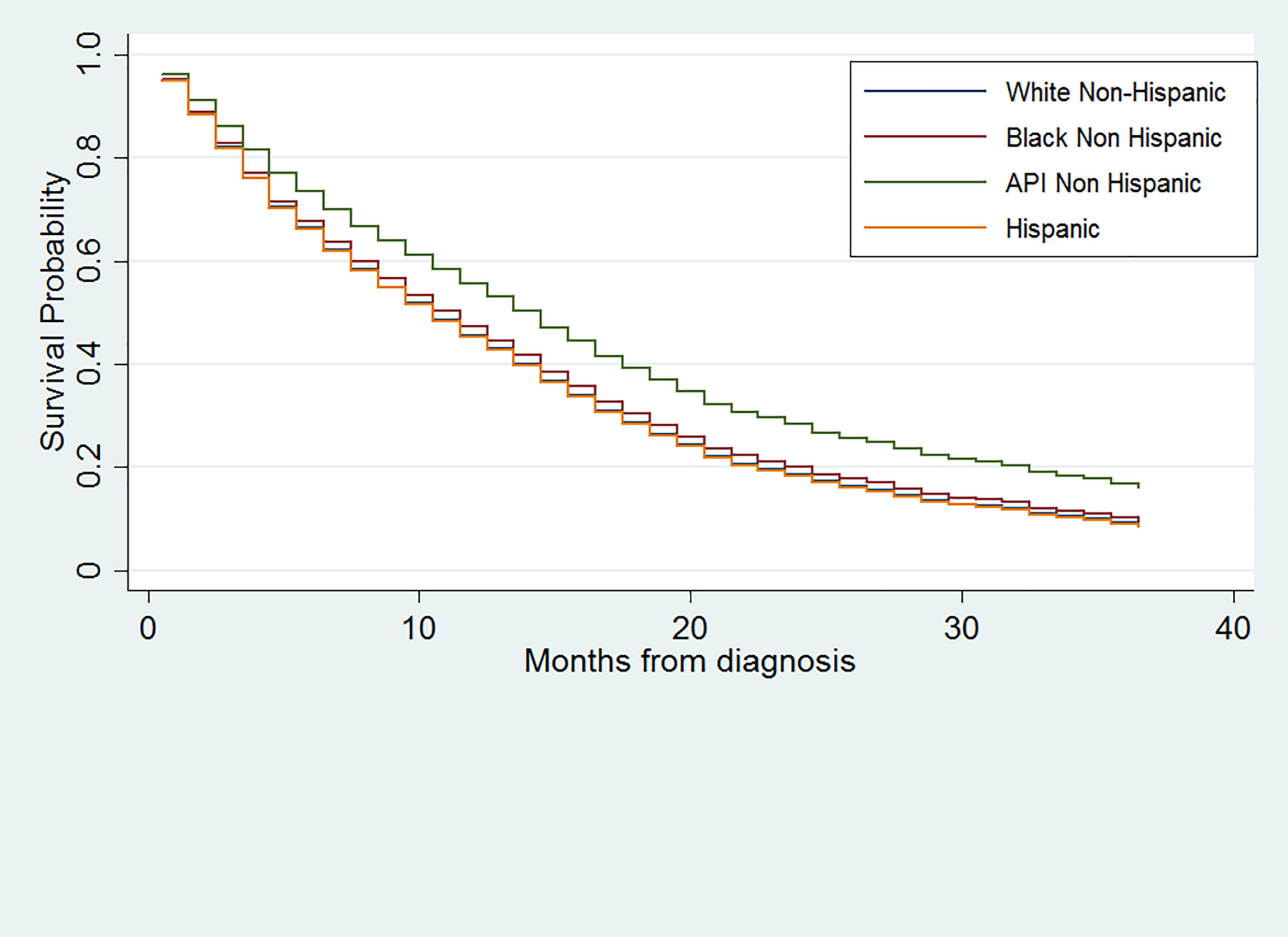
Adjusted survival estimates of the 3-year overall survival of glioblastoma patients by race/ethnicity; SEER 2010–2014.

The 3-year survival curve derived from Cox model (adjusted for age, gender health insurance, tumor site, tumor size, extent of the surgery) showed a better 3-year overall survival for API NH patients compared to Whites NH, Black NH and Hispanics (Fig 2).

## Discussion

This study found that API NH patients have a significantly better survival rate than White NH after controlling for age, gender, health insurance status, site, tumor size and extent of surgery. No significant differences in glioblastoma survival were observed among Black NH and Hispanic compared with White NH.

Our findings revealed no difference in survival between black and white GBM patients are in line with most of the previous population-based studies [7–10]. Only two previous studies reported differences in survival between white and black GBM patients [5, 15]. Barnholtz-Sloan and colleagues revealed, after adjusting for age at diagnosis, histological classification, and treatment modality, that blacks had a 13% increased risk of death per month compared to white patients [5]. The study by Shaw et al only included patients aged ≥70 years with glioblastoma as the first primary cancer diagnosed from 1999 to 2010 [15].

Studies including Asians are rare and revealed inconsistent results [7, 9, 10]. In agreement with our findings, Pan and colleagues reported that Asians had a statistically significantly lower risk of survival when compared with non-Hispanic whites [7]. Furthermore, a study in a large population study of 21,184 glioblastoma patients during 2001 and 2011 seemed to confirm the reduced risk of mortality of Asian patients in the US [10]. In contrast to their and our findings, the work by Farah et al showed an increased one-year survival, but not 5-year survival in Asian GBM patients. However, their risk estimates were not adjusted for covariates and rather descriptive in nature [9].

The scant evidence in regard survival of Hispanic GBM patients is in disagreement with the results of our study [7, 9, 10]. Pan et al revealed a statistically significant 13% risk decrease compared with white GBM patients [7]. It has to be kept in mind that Pan and colleagues used different histological definitions, including both giant cell GBM and gliosarcoma in their study whereas ours only included GBMs [7]. These tumor subtypes are significantly rarer and behave differently from GBM [17, 18]. For example, these two subtypes affect males and females at different rates than GBM, and they tend to have shorter survival times [17, 18]. Including these patients might affect the results, as they do not accurately represent the majority of GBM patients. A recent study, using data from 21,184 glioblastoma patients during 2001 and 2011 in adult Latino Americans also reported an increased survival for Hispanics compared with white non-Hispanics after adjustments for age at diagnosis, the extent of surgery, and differences in the use of radiation therapy [10]. However, they did not control for tumor size, sex or health insurance (a proxy for socioeconomic status) unlike ours.

In agreement with previous studies, we found that age at diagnosis was associated with survival in GBM patients [3, 19]. Whereas, Pan et al reported a five times higher hazard of death in the GBM patient population 75 years and older compared to the 19-34-year-old GBM patient group, Ohgaki et al revealed that younger patients survived significantly longer, ranging from a median of 8.8 months (<50 years) to 1.6 months (>80 years) [3, 19]. There are a few reasons that could account for this difference between age groups. First, it is possible that older patients have more comorbidities than their younger counterparts do. This overall worse state of health could make older patients less able to handle the insult of cancer. Secondly, older patients are enrolled in research trials disproportionately less, compared to their younger counterparts [20, 21]. Then, they are likely missing on newer possibly more effective treatment modalities. Finally, elderly patients, with less life expectancy than younger patients, are less likely to receive aggressive treatment. Pan et al., also found that radiation improved mortality, as did the degree of surgical intervention [3]. Consistent with this, patients receiving partial resection survived longer than those receiving no surgery, and gross total resection improved survival more than partial resection.

It is not clear whether the difference in survival between races is to due differences in genetic factors, environmental exposures, or differences in treatment. However, it has been suggested that possible differences in the molecular and genetic makeup of tumors between races, which might account for some of the discrepancies seen in mortality [17]. Wiencke and colleagues found that secondary glioblastomas have more TP53 mutations and that these types of tumors are found more often in black and Asian patients [18]. Conversely, Whites are diagnosed with more primary glioblastomas, which have been found to have more EGFR amplification [18]. As both secondary glioblastomas and TP53 mutations have been shown to have a better prognosis with a longer survival time as compared to primary glioblastoma and EGFR amplification, this could be one potential reason that blacks and Asians with glioblastoma may live longer than whites [2, 18]. Moreover, it is possible that by having a primary brain tumor before transformation into GBM, Asians and blacks might have earlier detection due to earlier symptomatology, closer follow-up, and more brain imaging.

Naturally, our study had some limitations. The database of SEER did not collect information on comorbidities that could possibly influence mortality. Furthermore, we were unable to adjust our analysis on patients’ extent of radiation therapy, including dosage and type, as the SEER database did not include that information anymore. It has been shown that the SEER registry data, collected by routine methods, may not be an appropriate source for documenting rates of radiation therapy receipt by breast cancer patients or for investigating geographic variation in radiation therapy receipt [22]. Previous studies have shown decreased completion of radiation therapy in the black population, and this could potentially be clouding our ability to detect whether blacks have a higher proportion of patients who fail to complete radiation therapy, as this has been shown to lead to a decreased mortality [21]. In addition, the database did not include information regarding chemotherapy or salvage therapy data, leaving surgery as the only possible treatment variable included in the analysis. The available data on documentation regarding surgery does not include volumetric data. This led us to include biopsy and subtotal tumor resection in the same group. Finally, despite some limitations, the SEER is an important population-based resource for understanding the implications of pathology diagnoses across demographic groups, geographic regions, and time, and provides unique insights into the practice of oncology in the U.S that are not attainable from other sources [23].

We would like to point out that the population used in our research was large and representative of roughly one-fourth of the United States population. Patients with multiple different primary cancers, those who have had previous cancers, those with extracranial GBM, and those with different subtypes of GBM; giant cell GBM and gliosarcoma were excluded. By excluding these patients, we got a better idea of the association typically found in patients with GBM.

In conclusion, our study identified the existence of a difference in survival in GBM patients based on race/ethnicity. API NH patients with glioblastoma have a better survival when compared to White NH patients, after controlling for age, gender, insurance status, site, tumor size and extent of surgery. Our findings expand on the topic of how race specifically affects a patient’s mortality, by using a population that is more generalizable to the typical GBM patient. Future prospective studies may investigate the cause of the differences in survival between API NH and other race/ethnicity groups. The association between EGFR and TP53 mutations and mortality, as well as the association between primary and secondary GBM and survival, may be studied independently of race, as this might be a key factor behind the differences we found. The findings from our study can help increase the accuracy of the prognostic outlook for white, black, Hispanic and API patients with GBM.
